# Tissue Viability of Free Flaps after Extracorporeal Perfusion Using a Modified Hydroxyethyl Starch Solution

**DOI:** 10.3390/jcm9123929

**Published:** 2020-12-03

**Authors:** Christian D. Taeger, Oliver Friedrich, Raymund E. Horch, Caroline Distler, Annika Kengelbach-Weigand, Carina Wenzel, Lukas Prantl, Konstantin Präbst

**Affiliations:** 1 Department of Plastic and Hand Surgery, University Hospital Regensburg, 93051 Regensburg, Germany; carina.wenzel@ukr.de (C.W.); lukas.prantl@ukr.de (L.P.); 2 Institute of Medical Biotechnology, Friedrich-Alexander University Erlangen-Nürnberg (FAU), 91054 Erlangen, Germany; oliver.friedrich@fau.de; 3 Department of Plastic and Hand Surgery, University Hospital of Erlangen, Friedrich-Alexander University Erlangen-Nürnberg (FAU), 91054 Erlangen, Germany; Raymund.Horch@uk-erlangen.de (R.E.H.); caroline.distler@mail.de (C.D.); Annika.Kengelbach-Weigand@uk-erlangen.de (A.K.-W.); 4 Institute of Bioprocess Engineering, Friedrich-Alexander University Erlangen-Nürnberg (FAU), 91054 Erlangen, Germany; konstantin_praebst@gmx.de

**Keywords:** hydroxyethyl starch solution, perfusion solution, extracorporeal perfusion, ischemia, free flap transfer

## Abstract

Background: In free flap surgery, tissue is stored under hypothermic ischemia. Extracorporeal perfusion (EP) has the potential to extend storage time and the tissue’s perspective of survival. In the present study, the aim is to improve a recently established, simplified extracorporeal perfusion system. Methods: Porcine *musculus rectus abdominis* were stored under different conditions. One group was perfused continuously with a simplified one-way perfusion system for six hours, while the other received only a single flush but no further treatment. A modified hydroxyethyl starch solution was used as a perfusion and flushing solution. Vitality, functionality, and metabolic activity of both groups were analyzed. Results: Perfused muscles, in contrast to the ischemically stored ones, showed no loss of vitality and significantly less functionality loss, confirming the superiority of storage under continuous perfusion over ischemic storage. Furthermore, in comparison to a previous study, the results were improved even further by using a modified hydroxyethyl starch solution. Conclusion: The use of EP has major benefits compared to the clinical standard static storage at room temperature. Continuous perfusion not only maintains the oxygen and nutrient supply but also removes toxic metabolites formed due to inadequate storage conditions.

## 1. Introduction

Preventing ischemia-related cell damage is a key challenge when preserving tissue extracorporeally for transplantation or replantation [1,2,3,4]. Presently, ischemically stored tissue is routinely kept under hypothermia to postpone ischemic damage [5] but the tissue’s time of survival is still very limited, especially if muscle tissue is involved [6]. Although in reconstructive surgery, a prolonged time of ischemia of free flaps is uncommon, any method that reduces even early ischemia-related cell damage is highly advantageous. This is particularly true in cases of reimplantation of amputates [7,8]. Even short-term exposure to ischemia can threaten the surgery’s success since it can result in effects like the “no reflow phenomenon” or “ischemia–reperfusion syndrome”, with the severity of these incidents being in direct correlation to the time of the preceding ischemia [9,10,11].

Extracorporeal perfusion (EP) has the potential to substantially increase the time of survival of tissue disconnected from blood circulation [12,13,14,15,16,17,18,19,20,21,22,23,24,25,26,27]. This is especially true in the case of major amputates since the separated limb contains a lot of muscle tissue that is very sensitive to ischemia, limiting the time of survival to 4 h [6]. In two clinical cases, we were able to perform a reconnection of amputated limbs after 12 and 15 h of extracorporeal storage. In these cases, an immediate replantation was not possible due to the patients’ critical medical condition. EP helped to bridge the time until both patients were stable enough for replantation. In these cases, we did not have to follow the principle of “life before limb” but were able to maintain both life and limb. [25]. Nonetheless, it still has no relevance in daily clinical routine [28]. One of the reasons is the high cost-to-benefit ratio. Most EP systems consist of a complex technical setup and demand a high level of expertise. To make EP a simple, cost-effective, yet powerful tool in cases of free flap transfer, we recently introduced a simplified EP system [24,27]. By omitting pumps and all forms of perfusate renewal, the perfusate is merely drained into the muscle from an infusion bag, while the outflow is discarded. We were able to demonstrate that the conservation of free muscle tissue in this system is comparable to a complex perfusion system with pump-mediated flow rate control and perfusate regeneration. The functionality and viability of perfused muscles using both EP systems show similar results, and they are both superior to ischemically stored muscles [24,27].

Nevertheless, there is still room for improvement, mainly the extension of the maximum time of storage. In the present study, we improved pre-existing results by modifying the perfusion solution. A colloidal solution was chosen to reduce edema formation, one of the major drawbacks of EP with acellular perfusion solutions [23,24,26,27,29]. The solution was then modified to improve the maximum storage time.

## 2. Methods

### 2.1. Animals, Surgical Protocol, and Experimental Setup

Animals (mature male pigs, n = 5; Erzeugergemeinschaft Franken Schwaben, Tierische Veredelung, Wertingen-Geratshofen, Wertingen, Germany) were treated identically to previous studies [22,23,24,26]. The surgical procedure was performed by a plastic surgeon, while anesthesia and viability parameter observation were conducted by a veterinarian. The detailed surgical method has already been published [22,23,24,26]. In short, the anterior rectus sheath was opened after a longitudinal incision over both *rectus abdominis* muscles in a supine position. The muscles were then dissected step by step from the anterior and posterior rectus leaf, and the deep inferior epigastric pedicle was dissected caudally as a pedicle vessel. At the end, a classical free *rectus abdominis* muscle flap was harvested bilaterally. All experiments were approved by the Government of Mittelfranken, Germany (No. 65-2532.2-65 1/10), and the Animal Care Committee of the Friedrich-Alexander University, Erlangen-Nürnberg. All experiments were carried out according to the relevant guidelines and regulations.

Both *rectus abdominis* muscles were used in the perfusion experiments. After the surgical procedure, the caudal segment of both muscles was cut between the third and fourth intersection, with the deep inferior epigastric pedicle accessible for cannulation (arterial: 22 G, d_i_ = 0.9 mm, l = 25 mm; venous: 18 G, d_i_ = 1.3 mm, l = 45 mm). Both muscles were flushed with 20 mL of perfusate (see Table 1) prior to storage experiments to remove intravasal blood residues, approx. 20 min before experiment initiation. One muscle was perfused continuously for six hours, while the other muscle did not receive any further treatment for the same duration (ischemic storage) at room temperature.

The perfusion solution was modified to improve storage conditions and, thus, increase the maximum storage time. A colloidal solution (Volulyte 6%^®^, Fresenius Kabi, Bad Homburg, Germany) was chosen as the base solution. Glucose (5 mmol/L) and calcium phosphate (2.3 mmol/L) were added to adjust the concentration to a physiological level, with the aim of enhancing the muscles’ ability to contract. Heparin (5000 IU/L) was administered to prevent the clotting of possible blood residues, and the pH was adjusted to a physiological level of 7.4.

**Figure 1:**
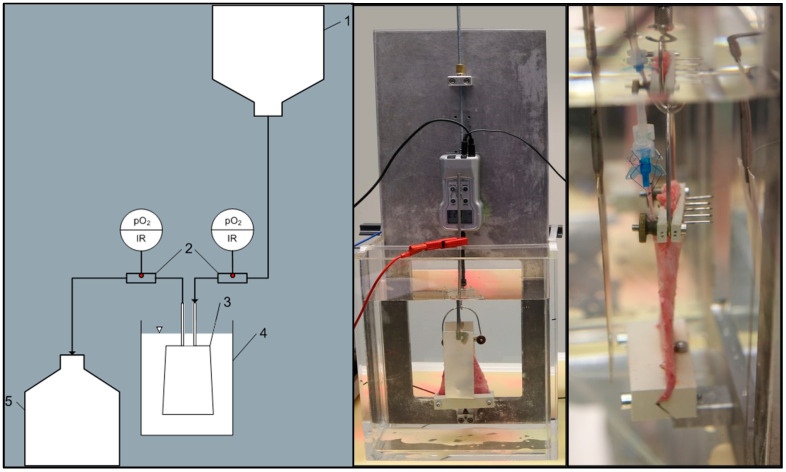
(**left**) Schematic representation of the experimental setup: reservoir bag (1), oxygen sensors (2), muscle flap (3) submerged in the reservoir (4), discarded perfusate (5). (**middle/right**) Pictures of the submerged muscle connected to a force gauge (**middle**) and the perfusate via cannulas (**right**).

Both muscles, perfused and ischemic, were clamped at the proximal end to the bottom of the EP system and connected to a force gauge unit by their distal end (see Figure 1). The muscles were then submersed independently in separate electrolyte solution (based on the composition of Jonosteril^®^) basins at room temperature. Oxygen consumption of the perfused muscle was calculated by continuous measurement of the perfusate’s arterial and venous oxygen content.

### 2.2. Extracorporeal Perfusion Parameters and Functionality Monitoring

The functionality of isolated muscle tissue is evaluated by its ability to exert force as a response to external electric field stimulation (EFS) [22]. This impulse is transmitted by two silver plate electrodes, placed at equal distance on each planar side of the muscle, and the electrolyte solution. The electrodes are connected to an external muscle stimulator, which generates a monopolar pulsatile square wave current (frequency 100 Hz, pulse width 1 ms). EFS is conducted every 15 min, with three measurements in direct sequence, each with an individual duration of 10 s; the resulting muscle force is then recorded. Muscles are initially stimulated with 0.5 V. Due to the detrimental effects of muscle damage or muscle exhaustion, the muscles’ forces decline over the duration of storage. To compensate for this decrease, the voltage is increased in steps of 0.5 V when the resulting force drops below a threshold of 0.25 N. Stimulating the muscles initially with a high voltage results in severe tissue damage.

The muscles’ forces are recorded with a force gauge connected to the muscles’ cranial end. Two resulting force levels can be observed over the ten-second measurement: a maximum force F_max_, which declines into a plateau over the recorded 10 s into a steady force F_steady_. The maximum force F_max_ serves as an indicator for an intact excitation-contraction coupling, while the steady force F_steady_ shows the muscle’s fatigue during ongoing stimulation [29].

To compare the forces from different muscles and to compensate for the increasing stimulation voltage, the forces F are normalized with reference to the stimulation voltage U_stim_. Furthermore, the forces are normalized with their initial force (F_0_) per voltage (U_stim,0_), resulting in an effective force:

Feff=qFUstimF0Ustim,0

The resulting effective forces (F_eff,max_ and F_eff,steady_) are plotted over the time of storage in the results. Effective force points were fitted with an exponential decay curve (using Sigma Plot v11.0 software, Systat Software, Inc., Berkshire, UK) to calculate the force decline rates (µ) as a comparative value for muscles under different storage conditions.

$$F_{\mathrm{eff}}=a\cdot e^{-\mu t}$$

The resulting force decline rates (µ) and coefficients of determination (R_sqr_) are summarized in the results.

### 2.3. Histology and Immunohistochemistry

Apart from force recordings, immunohistochemically stained histological samples were used to evaluate storage impact on cellular viability. Histological tissue sampling was reduced to two points in time, before and after extracorporeal storage, since tissue sampling affects perfusion and EFS measurements due to the injury of muscle fibers and blood vessels. The first sample was taken at the time of tissue harvest; the second was taken after six hours of storage.

All biopsies were fixed in 4% buffered formalin for 24 h and subsequently embedded in paraffin. The tissue’s viability was determined by the number of apoptotic cell nuclei highlighted with annexin V. Staining was performed using an anti-annexin V antibody (ab14196; Abcam, Cambridge, UK). The tissue samples, embedded in paraffin, were processed, cut, and stained according to previously published detailed protocol [22]. The slides were subsequently digitized (Panoramic-Midi and Panoramic Flash 250, 3D-Histech AG, Budapest, Hungary) to allow a standardized protocol for identifying the ratio of annexin-V-positive myonuclei to the total number of myonuclei: Φ_apoptotic_.

### 2.4. Perfusate Analysis

To evaluate the effectiveness of EP for muscle conservation, blood gas analysis (ABL800 Flex, Radiometer RSCH GmbH) was performed using venous perfusate samples. Glucose uptake, lactate formation, and the resulting changes in pH value, as well as potassium leakage, which indicates cell damage, were analyzed.

### 2.5. Statistical Analysis

A two-way analysis of variance (ANOVA) was carried out (with posthoc Holm–Sidak analysis) for values of F_eff_ with respect to the factor treatments (perfused, ischemic) and storage time using v11.0 Sigma Plot software (Systat Software, Inc.). For statistical analysis of Φ_apoptotic_ of perfused and ischemic muscles, a two-way analysis of variance (ANOVA) was carried out using the same software.

## 3. Results

**Figure 2:**
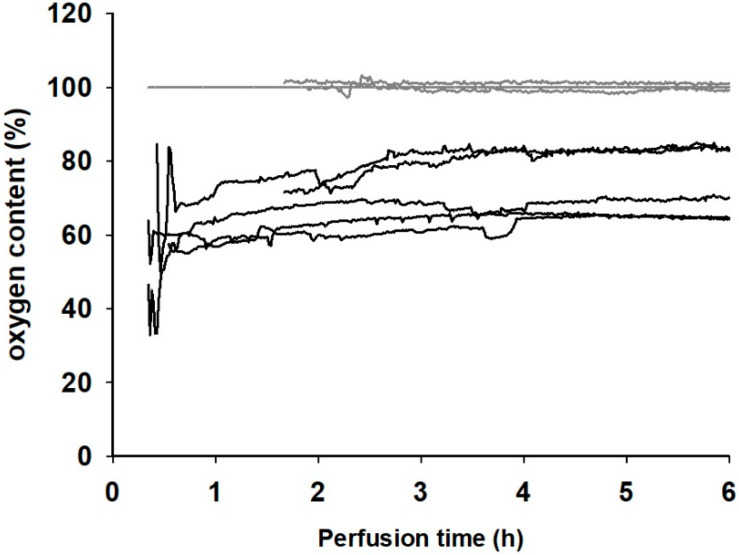
Oxygen content in % of the saturation concentration of the arterial (gray lines) and venous (black lines) perfusate.

With a fixed hydrostatic pressure of approx. 10 kPa, stable continuous perfusion of all muscles can be achieved, with an average perfusate flow rate of 0.70 (±0.23) mL/min. Oxygen consumption, calculated from the arterial and venous oxygen content of the perfusate solution (see Figure 2), the perfusion rate, and the muscles’ weight were within a physiological range, between 0.09 and 0.14 mL_O2_/min^−1^/kg^−1^. The perfused muscles developed interstitial edema during storage, resulting in an average weight increase of 49.4% (±5.9%). By definition, the ischemic muscles did not receive any form of perfusion and were, therefore, considered to be anoxic. Furthermore, they did not develop any edema.

**Figure 3:**
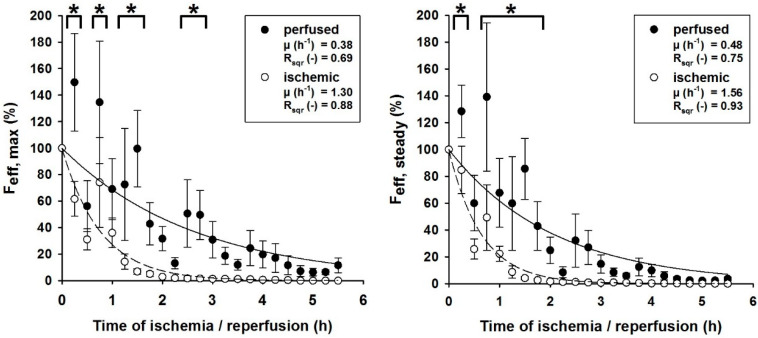
Normalized effective force per voltage of muscles undergoing hydrostatic EP (●) and ischemia (○) at different points in time after harvest; (**left**) effective maximum force (F_eff,max_) at different points in time; (**right**) effective steady-state force (F_eff,steady_) at different points in time. Mathematical fit of the form $F_{\mathrm{eff}}=a\cdot e^{-\mu t}$; µ: force decline rates; R_sqr_: coefficient of determination; error bars represent standard error of individual values recorded at the same time (n = 5); effective forces pooled under an asterisk (*) are statistically significantly different during the time of storage, indicating that EP is superior to ischemic storage during this time.

All perfused muscles presented a significantly higher rate of functionality compared to the ischemically stored muscles. While all perfused muscles were able to exert a force response after EFS, the ischemic muscles failed to do so between 3.5 and 5.25 h of ischemia. This behavior was also reflected by the force decline rates (µ) of the effective forces F_max_ and F_steady_ (see Figure 3). While the effective forces of perfused muscles declined with the rates of µ_max_ = 0.38 h^−1^ and µ_steady_ = 0.48 h^−1^, the forces of the ischemic muscle declined faster, with rates of µ_max_ = 1.30 h^−1^ and µ_steady_ = 1.56 h^−1^. Furthermore, the differences between the effective forces of both perfused and ischemic muscles were statistically significant up to 3.25 h of storage for F_max_ (*p* = 0.027) and 2.25 h for F_steady_ (*p* = 0.038).

**Figure 4:**
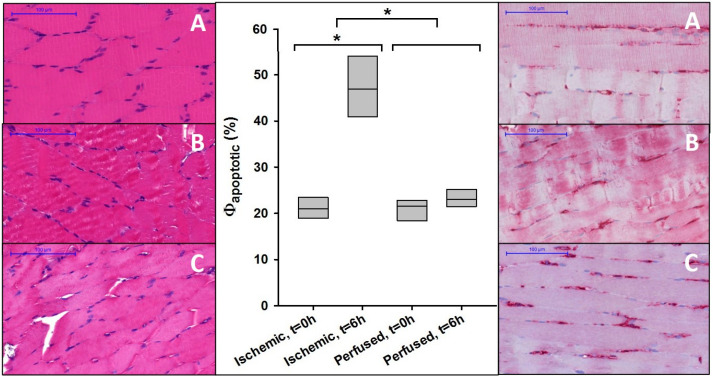
(**Left**) Histological images of the muscles prior to treatment (**A**), after six hours of ischemia (**B**), and after six hours of perfusion (**C**); (**middle**) ratio of annexin-V-stained cell nuclei in relation to the Table 0 h or groups (ischemic, perfused); (*) Asterisks indicate a statistical significance between values of different times (t = 0 and t = 6 h) or groups (ischemic, perfused); (**right**) histological images of the muscles with annexin V staining prior to treatment (**A**), after six hours of ischemia (**B**), and after six hours of perfusion (**C**); positively stained apoptotic myonuclei appear red, negatively counterstained myonuclei appear blue.

Apart from improved functionality, the perfused muscles also presented higher cellular viability. All samples of the ischemically stored muscles showed a statistically significant increase in ∆Φ_apoptotic_ = 26% after 6 h, indicating progressing apoptosis (see Figure 4). For all muscle samples of the perfused group, no significant increase in annexin-V-positive nuclei was observed after six hours, with an average increase of ∆Φ_apoptotic_ = 2.7%.

**Figure 5:**
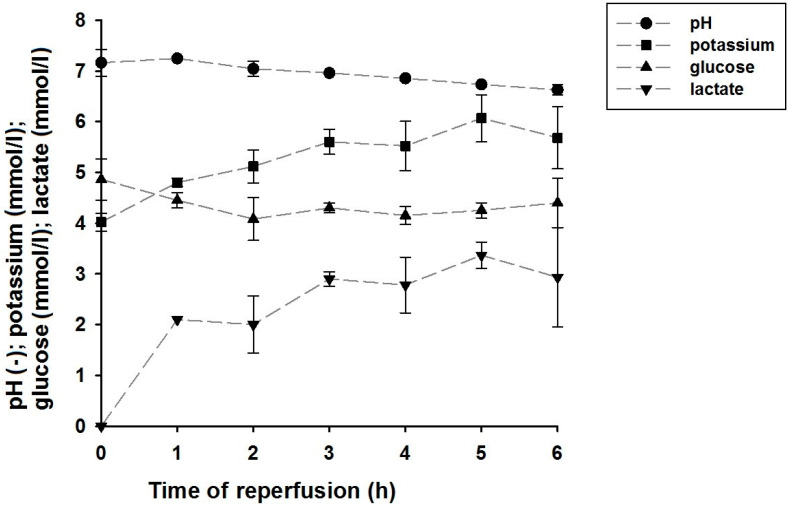
Blood gas analysis from the perfusate before EP (t = 0 h) and from the venous perfusate collected from muscles perfused with modified hydroxyethyl starch solution. Values were recorded at t = 0 h, measured with pure perfusate; all subsequent values were measured in samples collected from the venous drainage directly after the muscle’s cannulated vein. Dotted lines are included for better identification of the time course of data points.

Perfusate analysis also confirmed that the perfused muscle was metabolically active, as, for example, glucose was consumed, but it also indicated that the tissue’s cells were under stress. The pH decreased due to the formation of lactate, and an accumulation of interstitial potassium can be identified (see Figure 5). The data points at t = 0 h correspond to the composition of the perfusate (see Table 1). After six hours of perfusion, the pH of the perfusate dropped to a final value of 6.2 (±0.1). The glucose concentration dropped in the first two hours to a value of 4.1 (±0.4) mmol/L but slowly increased again to 4.4 (±0.5) mmol/L after six hours. Potassium concentration increased to a maximum of 6.1 (±0.5) mmol/L after 5 h of EP. After six hours, the potassium level was slightly decreased to a concentration of 5.7 (±0.6) mmol/L.

## 4. Discussion

Our intention is to develop a simple, cost-effective, and easily applicable perfusion system for the storage of free flaps and to make the benefits of this novel technique easily accessible in the clinical routine. In the present study, our aim is to improve our previously published method of a simple unidirectional EP [24] by using a modified perfusion solution.

The results indicate that EP is superior to ischemic storage. Perfused muscles show increased levels of vitality and functionality. While ischemically stored muscles show significant levels of apoptotic cells, perfused muscles do not. Furthermore, by extrapolating and comparing the force decline rates, perfused muscles, flushed with the modified starch solution, are functional up to 3.5 times longer than the ischemically stored ones. When compared to muscles stored using the common clinical standard static storage at room temperature after a flush with Jonosteril^®^ [22], the functionality can be preserved approximately 11 times longer with EP. This finding further stresses the importance of the storage solution.

The efficacy of EP as a tool to prevent ischemia-related cell damage in case of free flap surgery, as well as limb amputations, has been shown in several experimental approaches. Müller et al. were able to successfully reimplant porcine forelimbs after a period of 12 h of EP [20]. Wolff et al. (2016) described a series of three cases using whole blood for EP to achieve a free flap transfer without vascular anastomosis [30]. Hereby, EP was performed as a bridge until autonomization of the flaps occurred, and the flaps became independent from the external blood supply. However, using unidirectional perfusion requires a high amount of the patient’s blood. According to Wolff et al. (2016), EP was necessary for a successful free flap transfer since vessel-depleted necks left no possibility for vascular anastomosis. Being a well-established technique, even in the case of lower limb reconstruction with long distances [31,32,33,34], it is our opinion that it is implausible that the use of vascular loops or vein grafts in a neck reconstruction with very short distances would not be favorable to unidirectional EP with whole-blood.

The idea behind using blood as a perfusate for EP is obvious: the composition is inherently physiological, it provides physiological oncotic pressure, and it contains erythrocytes that increase the oxygen content of the perfusate. Nonetheless, studies using blood have reported lactate formation during EP as well, indicating oxygen deficiency [18,35,36]. Apart from the reported lactate formation, using blood as perfusate has some major disadvantages. First, blood availability is highly limited. Second, Fichter et al. (2016) suggested that the two major drawbacks of using blood as a perfusate are hemolysis and hemostasis [15], which can lead to the no reflow phenomenon. In preliminary experiments, perfusion with blood did not improve the outcome of EP [13]. Furthermore, in ischemic muscles, residual blood was still present even after flushing with perfusate. This supports the use of continuous perfusion without erythrocytes to avoid blood clotting and a subsequent “no reflow” phenomenon.

A promising approach was recently published by Kueckelhaus et al. (2017) using an acellular EP solution to preserve the forelimbs of pigs for a time period of 12 h, followed by heterotopic reimplantation [17]. EP perfusion was performed in a circuit using perfusate regeneration. This increases the complexity of the perfusion setup by including pumps, oxygenators, and bacterial filters to avoid contamination of the tissue, thus increasing equipment requirements and, consequently, time and investment. The resulting costs and efforts can render a complex EP system unfeasible. Slater et al. (2016) stated that the cost-to-benefit ratio is of major importance. The devices need to be small and mobile; otherwise, EP will not move into the clinical routine [28].

However, when using a unidirectional perfusion system without perfusate recycling, the amount of perfusate needed for continuous perfusion is not negligible. Nonetheless, discarding the venous perfusate provides an additional major advantage in that harmful metabolites, which accumulate in the perfusate during EP, are flushed out and not reintroduced into the tissue. With this in mind, removal of the perfusate is preferable to perfusate regeneration. Consequently, in our studies, only inexpensive perfusate solutions, which are nearly unlimitedly available, are used (Jonosteril^®^ and Volulyte^®^).

In conclusion, the use of EP has major benefits compared to the clinical standard static storage at room temperature. As a limitation, it has to be mentioned that although the flaps of our control group were kept under hypothermic conditions (i.e., room temperature), we have no data regarding perfusion with active cooling.

Continuous perfusion not only maintains oxygen and nutrient supply, if added, but also removes toxic metabolites caused by inadequate storage conditions. Therefore, even during short-term storage of muscle tissue below the critical ischemic time, EP helps to minimize the risk of ischemia-reperfusion damage and associated effects such as hemostasis. Even with a slight risk of edema-related complications, the consequences of ischemia-related damage are, by far, worse. The simplicity of the presented perfusion system, an infusion bag connected to a cannula, also allows for immediate application without special equipment or training. Despite these promising results, it should be noted that the necessity of extracorporeal perfusion is very rare and reserved for specific clinical scenarios.

## Figures and Tables

**Table 1:** Composition of the modified hydroxyethyl starch solution used as perfusion and flushing solution.

Component	Concentration
Na^+^	137.0 mmol/L
K^+^	4.0 mmol/L
Ca^++^	2.3 mmol/L
Mg^++^	1.5 mmol/L
CH_3_COO^-^	34.0 mmol/L
Cl^−^	110.0 mmol/L
C_6_H_12_O_6_	5 mmol/L
HES	0.5 mmol/L
Heparin	5000 IU/L
pH	7.4
